# Collargolum in Puerperal Infection

**Published:** 1908-02

**Authors:** 


### Collargolum in Puerperal Infection

E. BONNAIRE, Accoucheur to the Paris Hospital. (*Inter-* (*National Clinics*, Vol. III, 17th Series.) To obtain a slow, continuous and prolonged modifying action, collargol is used by inunction over a long period, or by the stomach, or by enema in 1 per cent. solution as has been done by Chrobak and Kuestner. But when an immediate and energetic effect is imperative, it must be injected intravenously. Collargol (1 per cent. to 10 per cent. solution) can also be used as a topic in localised infection, and gave us results superior to those from any other remedy in ophthalmia neonatorum. In purulent ophthalmia we obtained better results with 2 per cent. than with stronger solutions.

Except with patients who are practically dying, rise of temperature always occurs one or two hours after a collargol injection, reaching its maximum after the rigor. It only lasts a few hours and is followed, in the most favorable cases, by rapid defervescence or defervescence in lysis, the temperature becoming normal in two to six days if recovery is to be obtained by one injection. When further injections are necessary, hyperthemia takes place after each. The rise is sometimes considerable. In one instance, in which total defervescence followed in 36 hours, the temperature reached 108°.

Although less frequent, the rigor which accompanies or precedes the hyperthermia and the leukocytic manifestations is fairly regularly observed, particularly in favorable cases. In 40 cases of puerperal infection treated by collargolum at the Lariboisiere Hospital we have been struck with the relative frequency of the rigor in cases that recovered and its rarity in fatal cases. In 27 that ended in recovery, rigor was noted in 19, 75 per cent.; whereas it occurred but four times in the 13 that ended fatally, 30 per cent. This symptom therefore has prognostic value. It was absent in some patients who recovered, when the injection was made after a rigor due to the infection. It was always lacking in cases treated shortly before death. This rigor generally occurs only after the first injection, but we have observed it to be absent after the first and to appear after the following ones.

A third reaction, fully as common as the others, and to which all women themselves called attention, is the remarkable euphoria after an injection. Distressing symptoms—headache, intense thirst, dyspnea—disappear very rapidly. This was quite striking, for it took place in one patient at the same time as the rigor, while the temperature in the axilla rose to 108°. The rigor causes no more discomfort than that which follows normal delivery, and thus differs from septicemia chills.

Collargol gives rise to no signs of argyria (paralysis, nephritis, enteritis, etc., as is the case with silver nitrate. When we began to use collargol, we had just encountered a long series of failures with Marmorek's serum. In January, 1903, we made our first timid attempt, injecting 2 cc. of solution in a woman whose temperature oscillated between 100 and 104°, despite our endeavors. Defervescence was sudden and immediate, and was only disturbed secondarily by the appearance of an abscess. Since that day, taking only hospital cases into consideration, we have collected 40 complete observations. That this series of cases is so small, is due to our desire, until quite recently, to reserve collargol for the most serious cases—cases that resisted all ordinary methods of treatment—in order to collect reliable statistics on the value of collargol.

Twenty-seven or 70 per cent. made favorable recovery. In 15, cure was obtained with one injection, in 7 with 2 injections, and in 5 with 3 to 5 injections. Four of the cases had not proved refractory, but as they were grave from the start,—women with putrid feti and fever through labor or auto-contamination through extragenital suppuration,—collargol was injected at once, in a certain sense as a prophylactic. Of the 13 who succumbed, 9 received one injection, 3 of whom came to the hospital the day before death; 3 received 2 injections; and 1 died after long cachexia despite 5 injections. We desire to lay stress on the fact that in the majority of cases we turned to collargol as our last ray of hope.

